# A splicing-based multitissue association study of joint transcriptomes identified susceptibility genes for osteoarthritis

**DOI:** 10.3389/fimmu.2025.1590008

**Published:** 2025-09-11

**Authors:** Lantao Zhang, Fuxing Zhao, Hengheng Zhang, Xingbang Niu, Youliang Li, Chunxia Zhao, Jianhua Ding, Chaozheng Liu

**Affiliations:** ^1^ Department of Bone and Joint Surgery, Affiliated Hospital of Qinghai University, Xining, China; ^2^ Department of Breast Disease Diagnosis and Treatment Center, Affiliated Hospital of Qinghai University, Xining, China; ^3^ Department of Child Health Care, Qinghai Red Cross Hospital, Xining, Qinghai, China; ^4^ Department of Hematology, Wuwei Cancer Hospital of Gansu Province, Wuwei, Gansu, China

**Keywords:** osteoarthritis, transcriptome association study, LTBP1, single-cell sequencing, genetic susceptibility

## Abstract

**Background:**

Osteoarthritis (OA) is a common chronic degenerative joint disease worldwide, which seriously affects the quality of life of patients and adds economic burden. Although genome-wide association studies (GWAS) have identified multiple genetic loci associated with OA, the functional mechanisms of these loci remain unclear. Transcriptome association studies (TWAS) combining gene expression and GWAS data have provided new perspectives to explore the genetic basis of OA.

**Methods:**

This study integrated cross-tissue and single-tissue TWAS analyses as well as single-cell sequencing data to identify and validate the key genes associated with OA. Cross- and single-tissue analyses were performed using the UTMOST, FUSION, and MAGMA methods, while single-cell sequencing was applied for the investigation of the expression characteristics, pseudotemporal trajectories, and cell-to-cell communication patterns of the latent transforming growth factor beta binding protein 1 (LTBP1) in different cell subtypes.

**Results:**

This study identified multiple candidate genes associated with OA, among which LTBP1 displayed a significant association in both cross-tissue and single-tissue analyses (FDR < 0.05) and was validated as a key regulator of the transforming growth factor-beta (TGF-β) signaling pathway. Single-cell sequencing revealed that LTBP1 was differentially expressed in different chondrocyte subtypes and was associated with high enrichment of the Notch signaling pathway. Pseudotemporal analysis revealed the dynamic regulatory role of LTBP1 in chondrocyte differentiation.

**Conclusion:**

Intercellular communication analysis revealed that cells with high LTBP1 expression activated diverse signaling pathways such as TGF-β and vascular endothelial growth factor (VEGF), suggesting that it may be involved in the pathogenesis of OA by regulating the formation of the extracellular matrix and the immune response.

## 1 Introduction

Osteoarthritis (OA) is the most common chronic joint disease worldwide, with a significant increase in prevalence, especially among people aged ≥65 years, and its incidence of OA is 50-80% (1). The most common symptoms of OA are joint pain and dysfunction, which seriously affect patients’ quality of life (2). Past studies have demonstrated that the high prevalence of OA is associated with several risk factors, including age, obesity, gender (with a higher prevalence in women), joint injury, and genetic susceptibility (3, 4). OA poses a significant burden on individual patients and has a significant impact on national health systems and socioeconomics. According to past studies, the direct healthcare costs and indirect economic losses (e.g., work absenteeism and reduced productivity) owing to OA amount to billions of dollars (5). In some countries, OA is classified as one of the Class A chronic diseases, and the demand for its treatment has dramatically increased, especially in increasingly aging societies, indicating the need for future research and policies to focus more deeply on the prevention, early identification, and effective interventions for this disease (6).

The rapid development of transcriptomics technology in recent years has provided new means to explore disease mechanisms from the perspective of gene expression. Transcriptome-wide association studies (TWAS) are an approach for identifying genes associated with complex diseases whose genetic effects may be mediated through transcriptome (7). TWAS utilize reference genetic and transcriptome data to estimate the magnitude of the effect of genetic variation on gene expression (i.e., the effect sizes for broadly expressed quantitative trait loci, eQTL). These estimated effect sizes serve as variant weights in gene-based association tests and facilitate the mapping of risk genes to genome-wide association study (GWAS) data (8). For example, previous studies have identified multiple GWAS signals associated with OA, albeit the functional nature of these loci and specific associations with the OA phenotype remain unclear (9, 10).

This study aimed to investigate the gene expression patterns associated with OA in different tissues (e.g., articular cartilage, synovium, and bone tissues) using cross-tissue transcriptome association analysis and also study their roles in OA susceptibility. We have integrated multiple databases and bioinformatics tools with GWAS results to infer the changes in expression of key genes and explore their roles in OA pathogenesis. Through this integrated approach, we hope to provide new insights into the molecular mechanisms of OA and thereby provide a theoretical basis for future targeted therapeutic strategies.

## 2 Methods

### 2.1 Materials and methods

**Figure 1:**
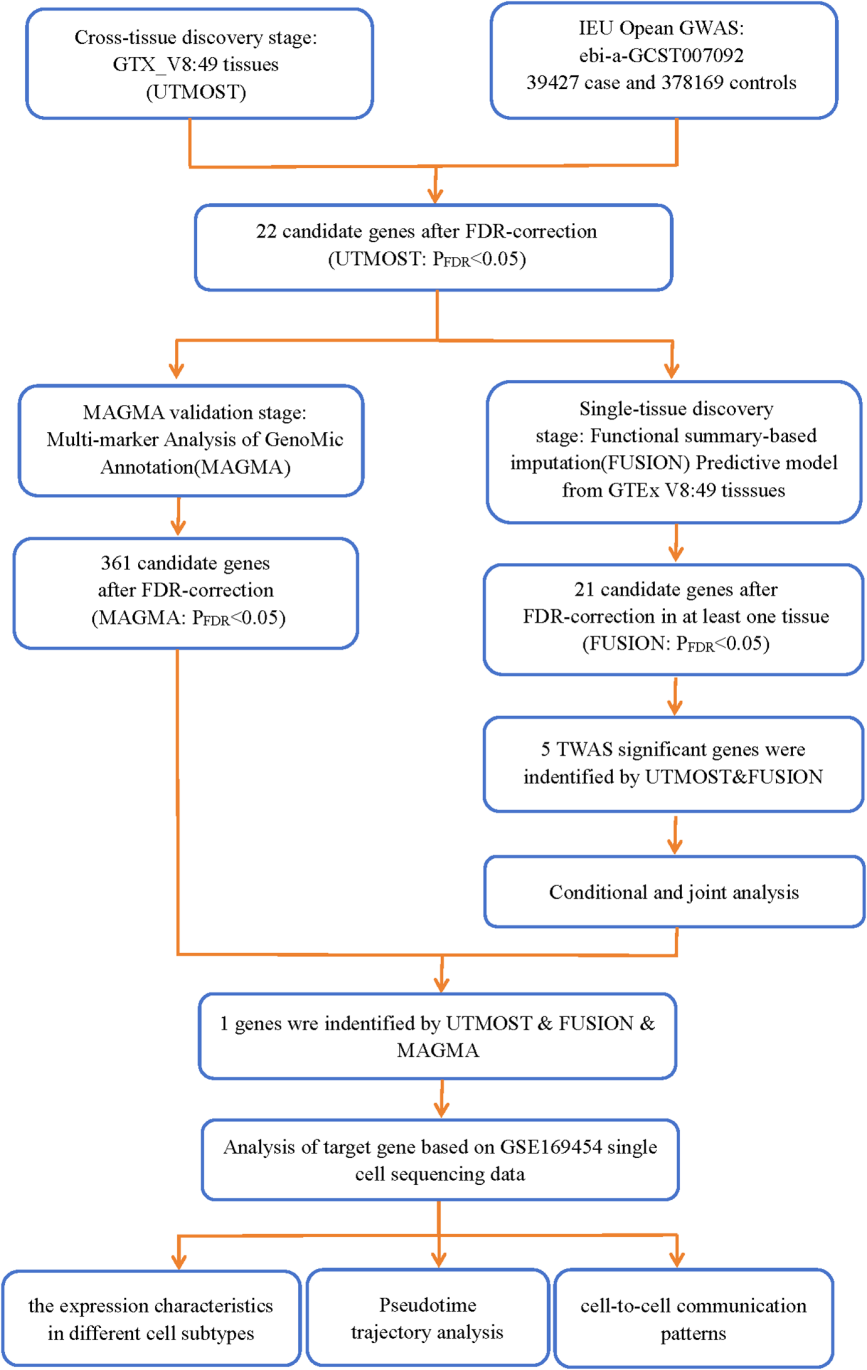
Analytical flowchart of this study.

The analysis process is depicted in **Figure 1**.

### 2.2 OA GWAS data sources

In this study, the osteoarthritis-related dataset was sourced from the IEU open GWAS database (https://gwas.mrcieu.ac.uk/datasets/ebi-a-GCST007092), which includes 39,427 cases and 378,169 European ancestry controls. The dataset originates from a large-scale genome-wide analysis conducted by Ioanna Tachmazidou et al. using data from the UK Biobank. The study aimed to identify new therapeutic targets for OA (11).

The gene expression data used in this study were obtained from public databases (e.g., GTEx and Tissue Atlas), covering a wide range of tissue samples, including articular cartilage, synovium, and bone tissues. We selected the GWAS dataset related to OA to ensure the analysis is relevant. This dataset included 39,427 cases and 378,169 controls of European ancestry.

### 2.3 Source of the eQTL files

The GTEx V8 dataset (The Genotype-Tissue (2013) Expression (GTEx) project. Nat Genet 456:580-585) contains extensive gene expression data from 49 different tissues.

### 2.4 Cross-tissue TWAS analysis

We used cross-tissue UTMOST analysis (https://github.com/Joker-Jerome/UTMOST?tab=readme-ov-file) to quantify the overall gene-trait associations at the organism level. This approach helps identify more genes with enriched trait heritability within a tissue, which, in turn, helps improve the accuracy of the estimation (12, 13). Subsequently, we integrated gene-trait associations using the generalized Berk-Jones test with single-tissue statistics of covariance (14). A significance level of false discovery rate (FDR) < 0.05 was considered to indicate statistical significance after applying a FDR correction.

### 2.5 TWAS analysis of single organizations

We performed TWAS analysis using the FUSION tool (http://gusevlab.org/projects/fusion/), which combines OA GWAS data with eQTL data from 49 tissues of GTEx V8 for the estimation of the association of each gene with the disease (15). Initially, the linkage disequilibrium (LD) between the prediction model and the SNPs at each locus of the GWAS was estimated by using 1,000 European genomic samples. Subsequently, FUSION was used to integrate several prediction models (such as BLUP, BSLMM, LASSO, Elastic Net, and Top 1) to assess the overall effect of SNPs on the gene expression weights. The model with the highest prediction performance was then utilized to determine the gene expression weights (16). Subsequently, we combined the genetic effect of OA (OA GWAS Z-score) with these gene weights for the OA TWAS study. The subsequent studies included candidate genes that met the following two criteria (1): FDR < 0.05 in the cross-tissue TWAS analysis (2); FDR < 0.05 in at least one tissue in the single-tissue TWAS analysis.

### 2.6 Conditional and joint analyses

FUSION allows the identification of multiple related features in a locus and determination of conditionally independent ones among those. Accordingly, we performed conditional and joint (COJO) analysis (a postprocessing module of FUSION) to identify independent genetic signatures (17). COJO analysis ensures a more comprehensive understanding of the genetic architecture of trait variation by accounting for LD among markers (18). After testing, genes representing independent associations were referred to as jointly significant genes, whereas those that no longer showed significance were considered marginally significant genes.

### 2.7 Gene analysis

For the gene analysis, we used the default parameters of the MAGMA software (version 1.08) to summarize the association statistics at the SNP level into gene scores, thereby quantifying the degree of association of each gene with the phenotype (19, 20). Detailed information on the parameter settings and a comprehensive methodology is available in the original MAGMA document (21).

### 2.8 Samples and data collection

The expression matrix and metadata files for the single-cell RNA sequencing dataset GSE169454 were obtained from the Gene Expression Omnibus (GEO, https://www.ncbi.nlm.nih.gov/geo/). This dataset includes 140,039 cells from 3 normal cartilage tissue samples and 4 osteoarthritic cartilage tissue samples.

### 2.9 Single-cell sequencing analysis

Data preprocessing and analysis were performed using the Seurat R software package. After importing the GEO dataset, quality control metrics were applied to remove low-quality cells based on the mitochondrial gene content and the total gene count. We used the harmony R package to normalize the data and correct for batch effects. Dimensionality reduction was performed using principal component analysis via the RunPCA function. A K Nearest Neighbors graph was constructed using the FindNeighbors function, and the cell clusters were identified using the FindClusters function via Shared Neighborhood modular optimization.

Differentially expressed genes (DEGs) between the cells with high and low latent transforming growth factor beta binding protein 1 (LTBP1) expression were identified using the FindMarkers function. Gene Set Variation Analysis (GSVA) was performed using the marker gene sets in MSigDB to assess activity of the pathway in the LTBP1 high-expression group. The pathway enrichment scores were calculated using the GSVA software package.

Cell differentiation trajectories were reconstructed using the Monocle software package to determine the developmental progression of OA cells. Intercellular communication was assessed using the CellChat software package, which determines cell-cell interactions based on known ligand-receptor pairs. Signaling pathways and communication networks were compared between the LTBP1 high- and low-expression groups.

## 3 Results

### 3.1 Cross- and single-tissue TWAS analysis

**Figure 2:**
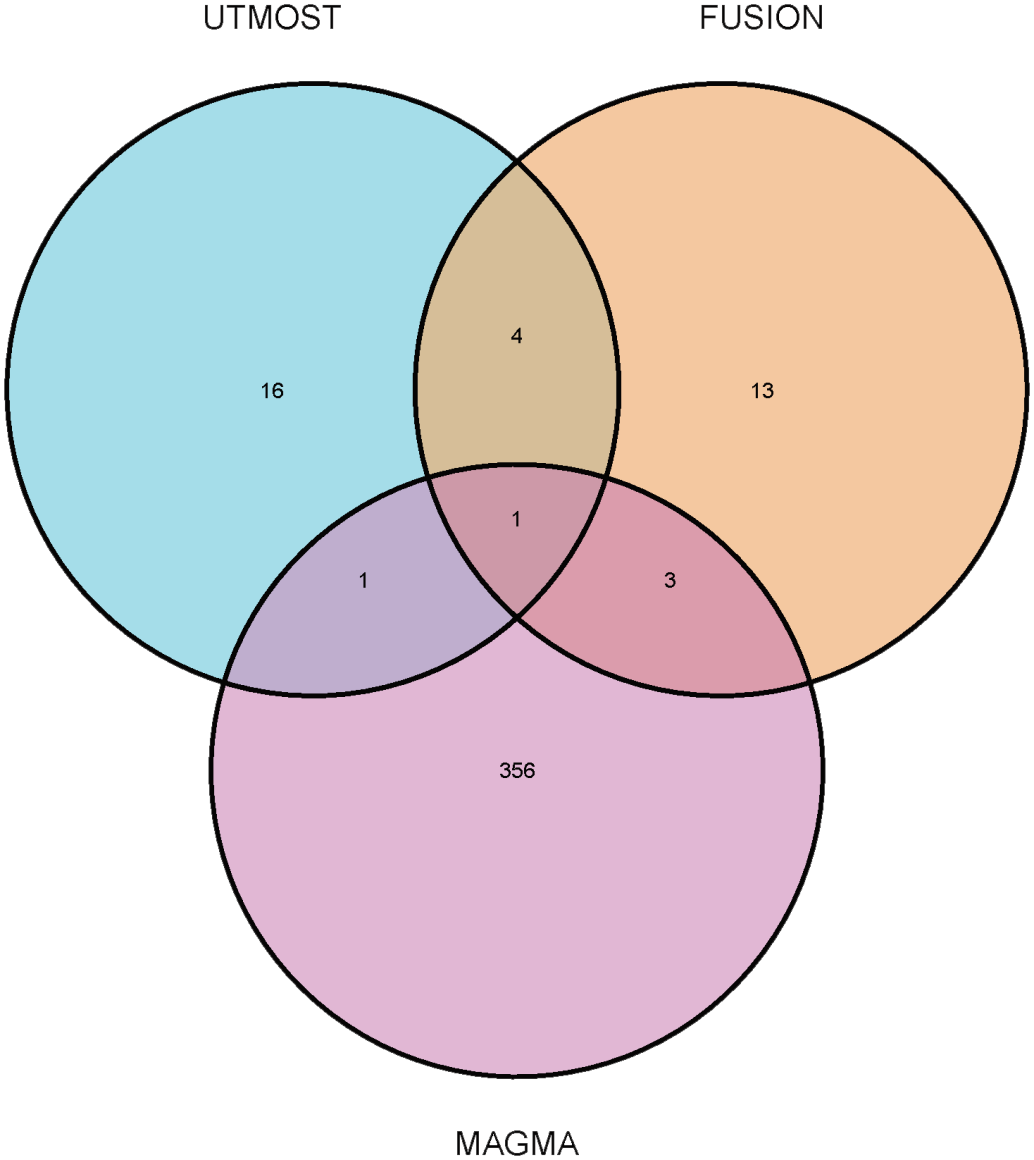
Venn diagram. MAGMA identified 361 significant genes associated with osteoarthritis, FUSION identified 21, and UTMOST cross-tissue analysis identified 22, of which 1 were common.

We performed cross- and single-tissue TWAS association analyses for OA using the UTMOST method, the FUSION tool, and the MAGMA gene annotation and validation method. Through UTMOST analysis, we identified 22 statistically significant candidate genes (FDR < 0.05) (**Supplementary Table 1**). These genes may play a crucial role in the pathogenesis of OA. Using the same 49 tissues, the FUSION model selected 21 candidate genes that were significant in at least one tissue (FDR < 0.05) (**Supplementary Table 2**). The MAGMA analysis, based on the multi-marker gene annotation model, identified 361 candidate genes (FDR < 0.05) (**Supplementary Table 3**). On combining the results from UTMOST and FUSION, we finally identified five statistically significant TWAS genes. In addition, LTBP1 was identified as significant in UTMOST, FUSION, and MAGMA analyses during the MAGMA validation phase and was particularly validated in the OA correlation analysis (**Figure 2**). This finding suggests that LTBP1 is a key pathogenic gene that warrants further functional validation and biological studies.

### 3.2 OA TWAS loci are driven by expression signals

**Figure 3:**
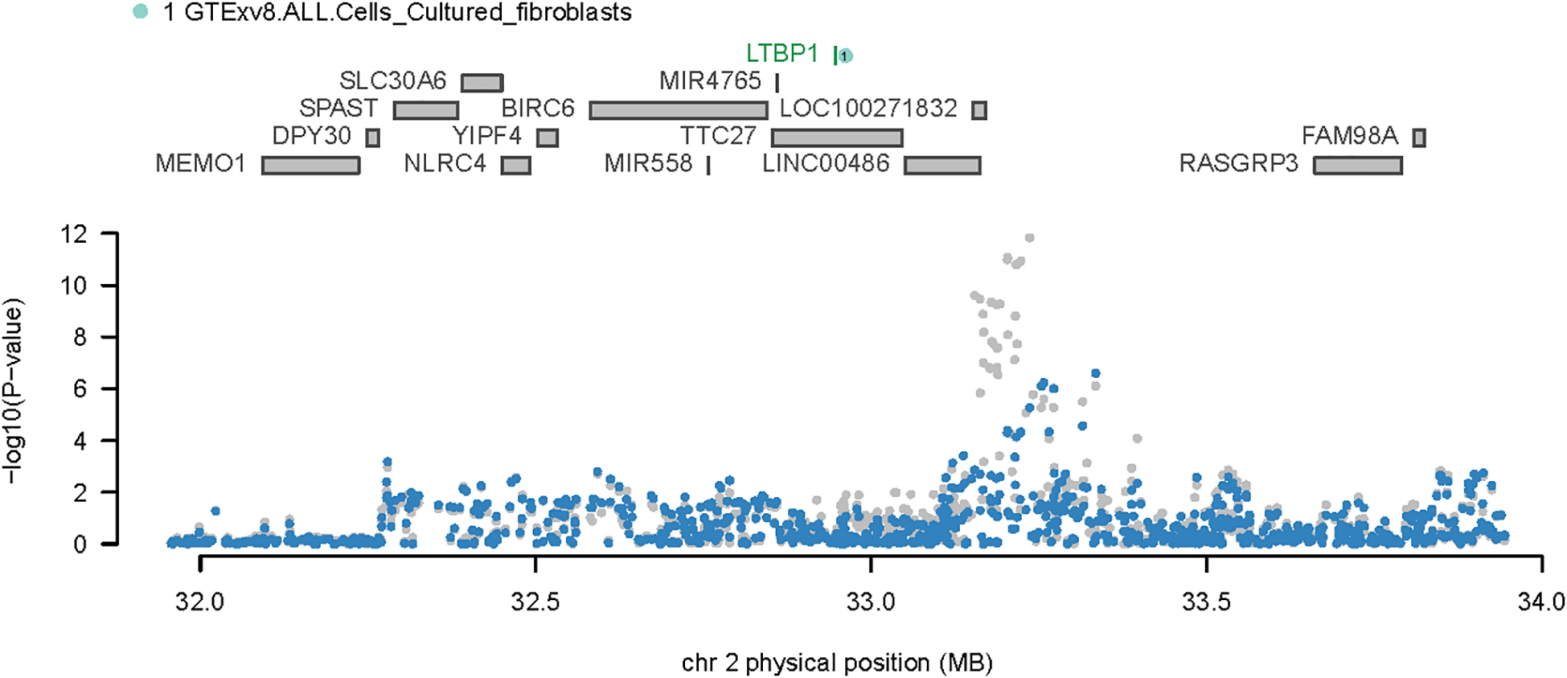
Regional association plot for chromosome 2. The upper portion of the plot displays all genes in the region, while the lower portion presents the regional Manhattan plot of GWAS data before (gray) and after (blue) the predicted expression regulation.

Considering the overlap between certain TWAS signals and significant OA loci, we performed COJO analyses to ascertain whether the associated genes were influenced by multiple related traits or represented independent conditions. Upon eliminating the effects of other gene interactions, LTBP1 was found to be responsible for all signals at its locus. Notably, SNP rs4630744, which guided the SNPGWAS, exhibited a strong association with OA (*P* = 4.62e-08); and this significance was upheld when conditioned on LTBP1 (*P* = 3.40032e-05) (**Figure 3**).

### 3.3 Functional analysis of the key genes

**Figure 4:**
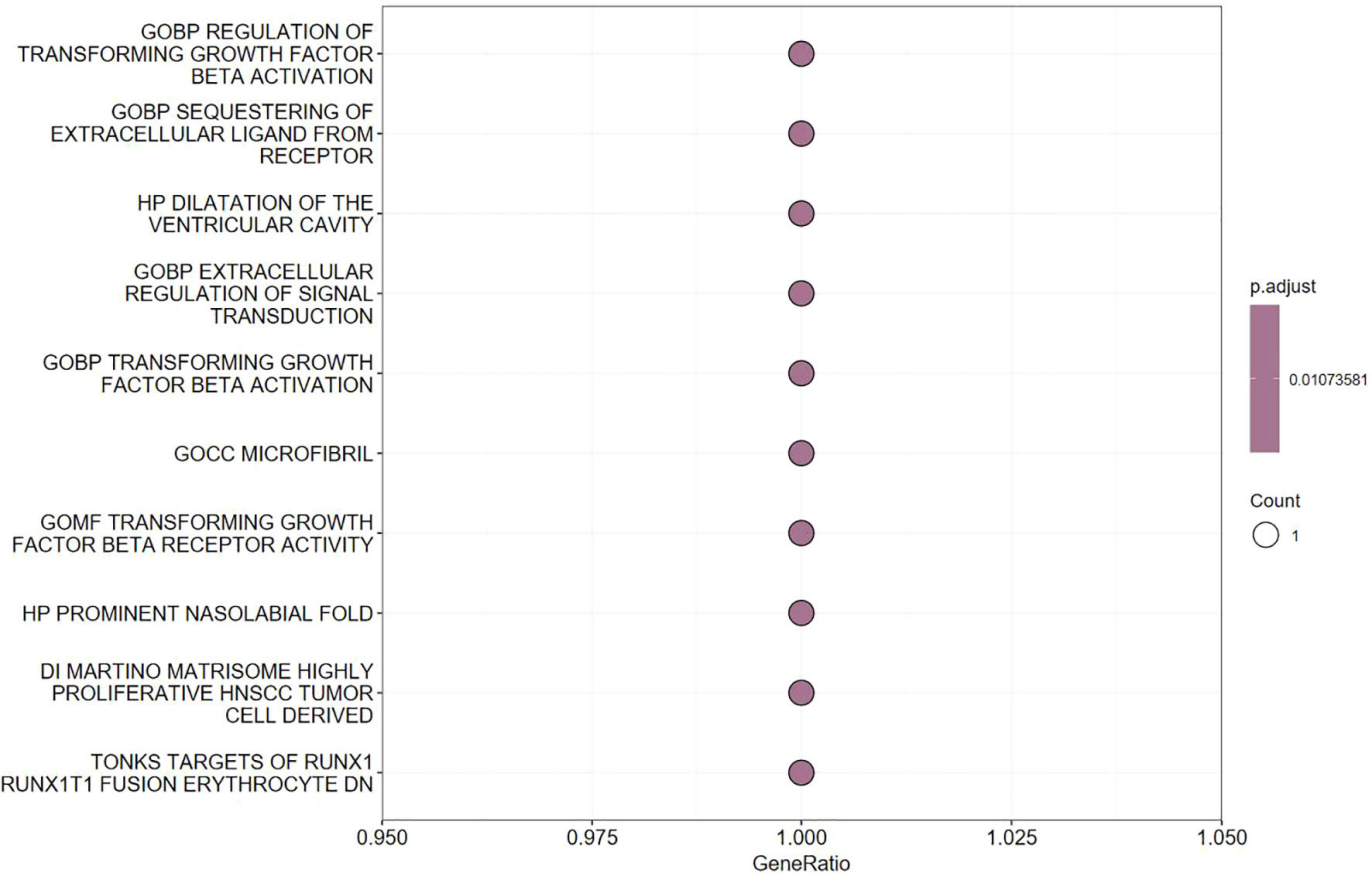
Pathway enrichment analysis of LTBP1 gene.

We specifically focused on LTBP1, as it is closely related to the OA pathogenesis. Functional enrichment analysis revealed that LTBP1 plays a key role in the transforming growth factor-beta (TGF-β) signaling pathway, which is associated with cell proliferation, cartilage repair, and immune regulation (**Figure 4**). This finding suggests that LTBP1 may influence OA progression by regulating the extracellular matrix (ECM) formation and cell growth pathways.

### 3.4 Molecular characterization of LTBP1 at the single-cell sequencing level

**Figure 5:**
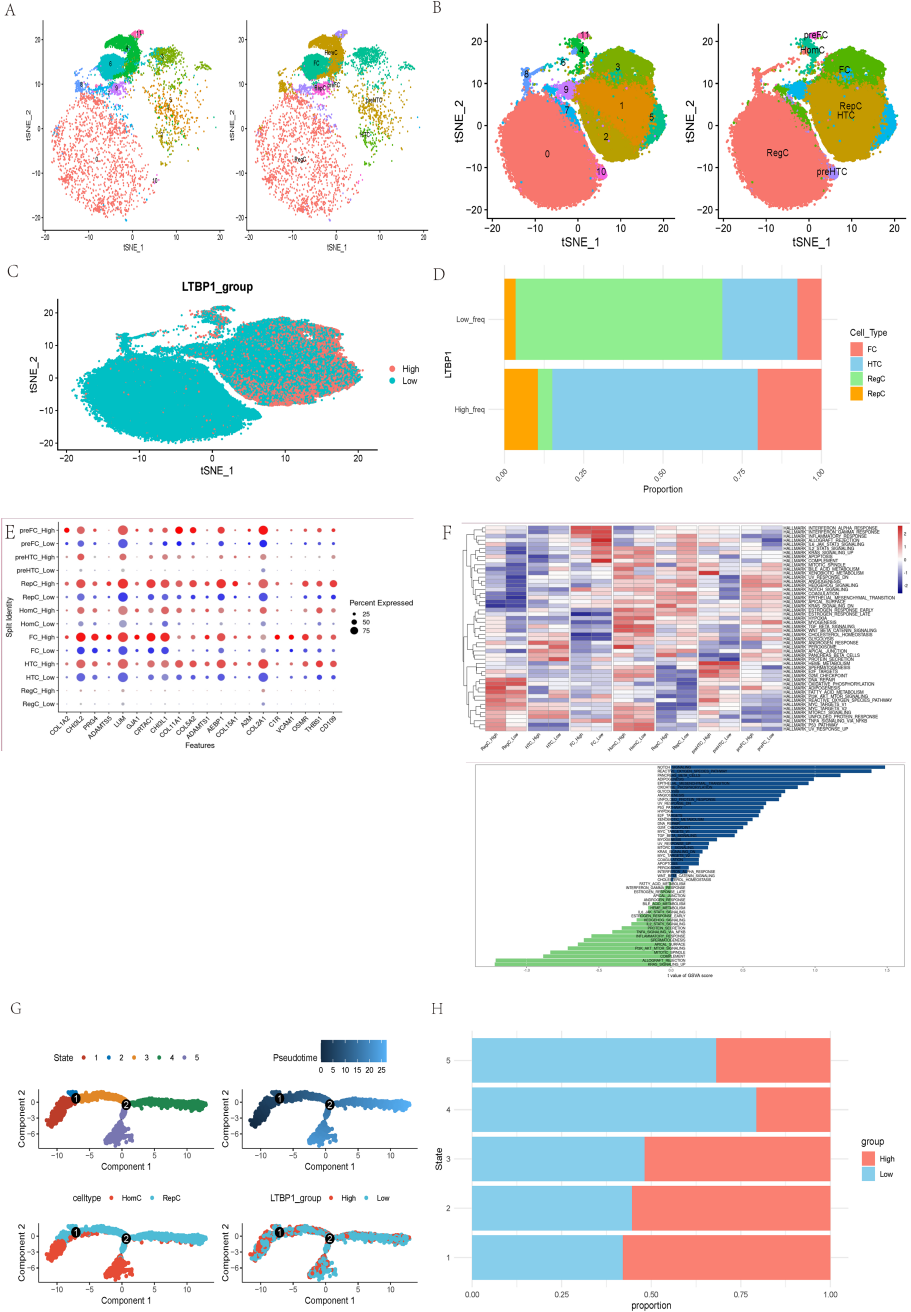
Molecular features of LTBP1 at the single-cell level. **(A)** t-SNE for the dimension reduction and visualization of normal tissues, and 7 cell types. **(B)** t-SNE for the dimension reduction and visualization of OA samples, and 7 cell types. **(C)** t-SNE for the dimension reduction and visualization of cells with high or low LTBP1 expression. **(D)** Relative proportion of four cell types in cells with high or low LTBP1 expression. **(E)** The differentially expressed genes among the identified 7 cell types at varying LTBP1 expression levels. **(F)** GSVA analysis of differentially expressed genes between high and low LTBP1 expression. **(G)** Pseudotime trajectory analysis of OA cells. **(H)** LTBP1 expression in five cell states based on pseudotime analysis.

We investigated LTBP1 characteristics in three normal and four OA samples using single-cell sequencing analysis. Seven cell types were identified in the normal (**Figure 5A**) and OA samples (**Figure 5B**). Results for LTBP1 expression are illustrated in **Figure 5C**. Regulatory cells (RegC) were associated with low LTBP1 expression, whereas fibroblast cells (FC), hypertrophic chondrocytes (HTC), and regenerative cells (RepC) exhibited high LTBP1 expression (**Figure 5D**). These terms refer to specific groups of cells in the cartilage environment, not subtypes of chondrocytes. Each cell playing a unique role in the pathogenesis of OA. DEGs among the seven cell types at varying LTBP1 expression levels are depicted in **Figure 5E**. GSVA analysis revealed the notch signaling pathway to be the most enriched signature in cells with high LTBP1 expression. The heatmap of the enriched pathways across different cell subtypes is illustrated in **Figure 5F**.

Pseudotime trajectory analysis, performed using the Monocle package, identified five cell states based on one branch point in OA cells. The cells transitioned from state 3 to states 4 and 5 with the progression of pseudotime (**Figure 5G**). Notably, the LTBP1 expression was highest in state 1 compared with that in the other states. Homogeneous cells (HomC) were predominantly in states 1 and 5, whereas RepC were in multiple cell states, 2, 3, and 4 (**Figure 5G**). LTBP1 expression levels varied across states, with a higher expression in states 1, 2, and 3 and a lower expression in states 4 and 5 (**Figure 5H**). The developmental trajectory and dot plot of the top 6 DEGs are shown in **Supplementary Figures 1A, B**, the heatmap of the top 50 DEGs in **Supplementary Figure 1C**, the developmental trajectories of different samples in **Supplementary Figure 1D**, and the developmental trajectory of LTBP1 in **Supplementary Figure 1E**.

### 3.5 Intercellular communication associated with LTBP1

We comprehensively analyzed the intercellular communication of OA cells with high and low LTBP1 expression. The seven cell types were categorized into the following four types based on functional roles: receiver, sender, mediator, and influencer. In the LTBP1 high-expression group, the communication patterns of the receivers (incoming signals) were categorized into five different types, whereas the communication patterns of the senders (outgoing signals) were categorized into four different types (**Supplementary Figures 2A, B**). The dot plots in **Supplementary Figures 2C, D** illustrate the communication patterns of the seven cell types, and the Sankey plots in **Supplementary Figures 2E, F** show that HomC were associated with the receiver pathway and RegC and FC with the sender pathway in pattern 2. In addition, all receiver and sender pairings are summarized in the dot plots in **Supplementary Figure 2G**.

In the LTBP1 low-expression group, the receiver and sender communication patterns were categorized into three different types (**Supplementary Figures 3A, B**). The communication patterns among the seven cell types were also visualized (**Supplementary Figures 3C, D**). Sankey diagrams indicated that HTC and FC were associated with the receiver pathway, and the HTC with the sender pathway in pattern 1 (**Supplementary Figures 3E, F**). The dot plots in **Supplementary Figure 3G** show all receiver and sender pairings.

**Figure 6:**
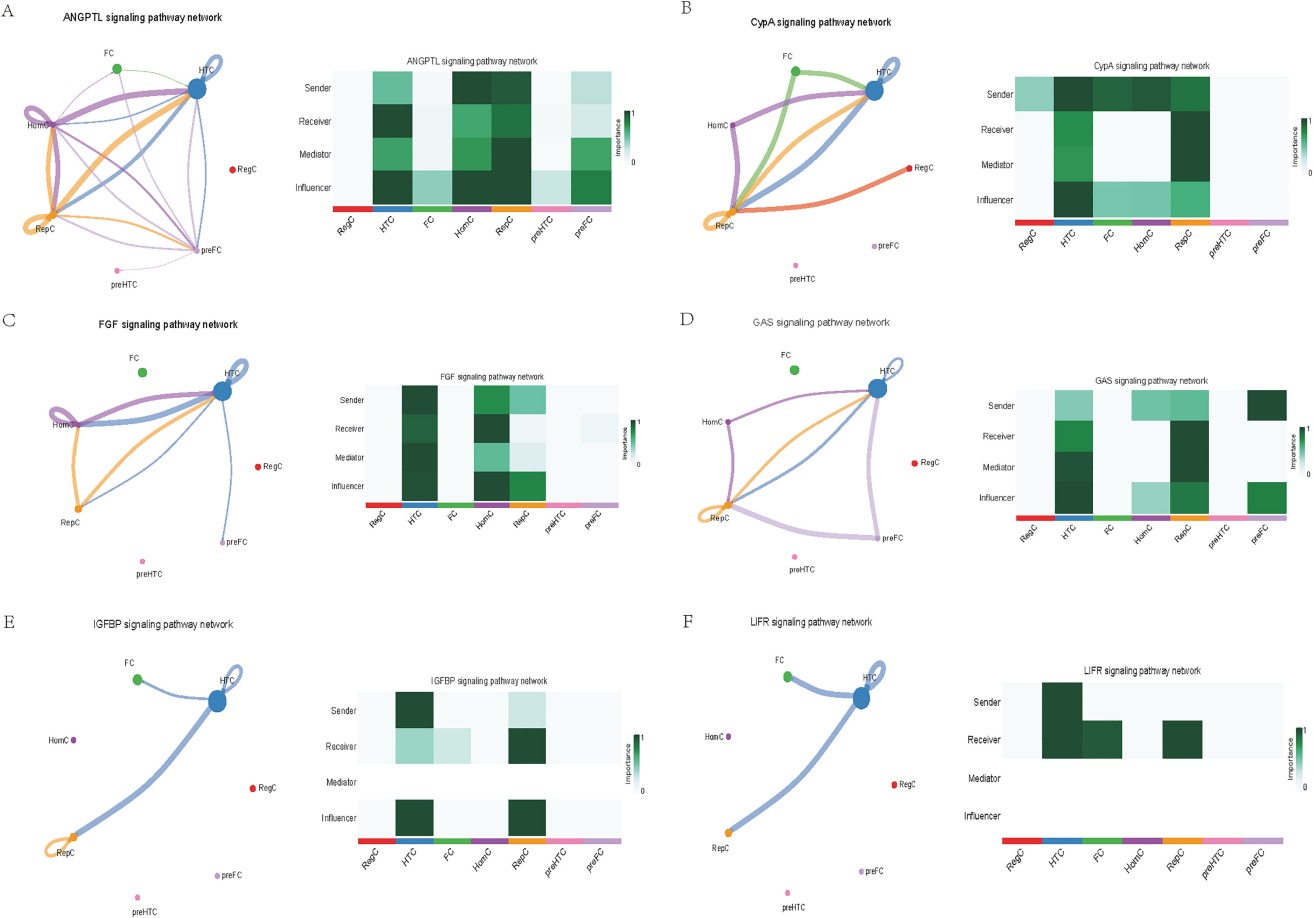
Cellular interaction of OA cells with high LTBP1 expression. The cellular interaction network identified cell clusters in various signaling pathways, including **(A)** ANGPTL, **(B)** CypA, **(C)** FGF, **(D)** GAS, **(E)** IGFBP, **(F)** LIFR signaling pathways.

**Figure 7:**
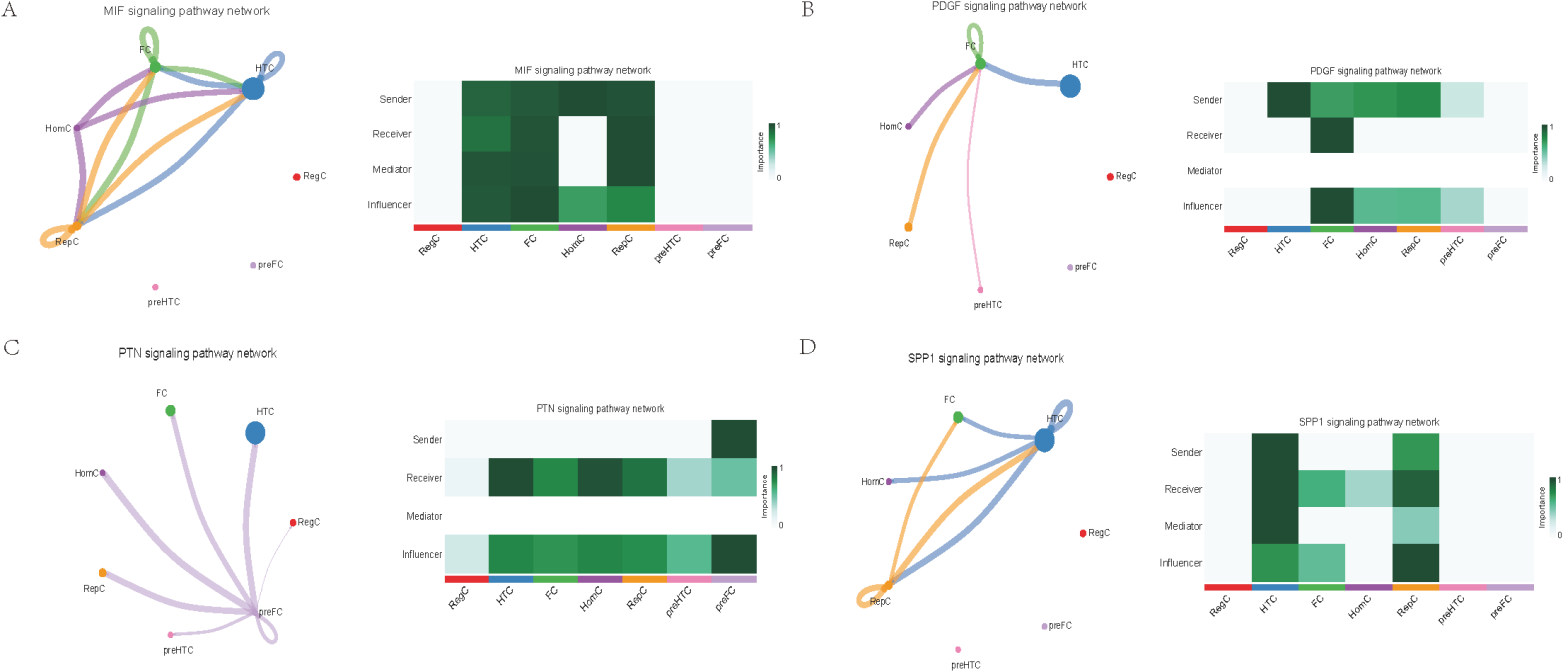
Cellular interaction of OA cells with high LTBP1 expression. The cellular interaction network identified cell clusters in various signaling pathways, including **(A)** MIF, **(B)** PDGF, **(C)** PTN, **(D)** SPP1 signaling pathways.

**Figure 8:**
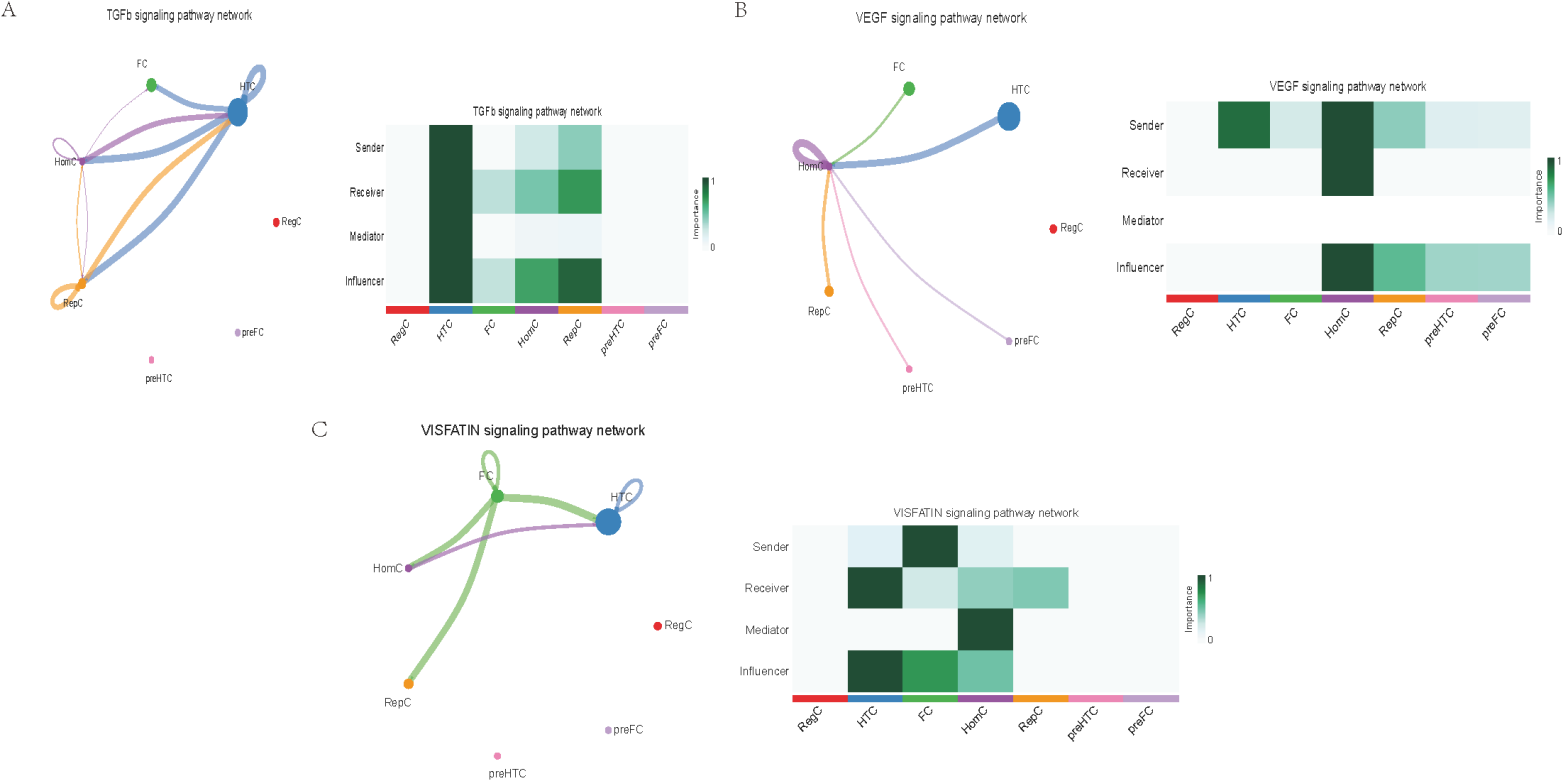
Cellular interaction of OA cells with high LTBP1 expression. The cellular interaction network identified cell clusters in various signaling pathways, including **(A)** TGFβ, **(B)** VEGF, **(C)** VISFATIN signaling pathways.

Further exploration of the association between LTBP1 expression and specific signaling pathways revealed that OA cells with high LTBP1 expression were predominantly activated by the angiopoietin-like protein (ANGPTL), cyclophilin A (CypA), fibroblast growth factor (FGF), growth arrest-specific protein (GAS), insulin-like growth factor binding protein (IGFBP), leukemia inhibitory factor receptor (LIFR), macrophage migration inhibitory factor (MIF), platelet-derived growth factor (PDGF), pleiotrophin (PTN), secreted phosphoprotein 1 (SPP1), TGF-β, vascular endothelial growth factor (VEGF), and visceral fat-derived adipokine (VISFATIN) signaling pathways (**Figures 6**–**8**). In contrast, OA cells with low LTBP1 expression predominantly activated the ANGPTL, CypA, FGF, IGFBP, MIF, SPP1, TGF-β, and VISFATIN pathways (**Supplementary Figure 4A-H**).

## 4 Discussion

In this study, we systematically explored the genes associated with OA susceptibility and the underlying molecular mechanisms by combining cross-tissue transcriptome association analysis (UTMOST), single-tissue analysis (FUSION), and gene annotation and validation analysis (MAGMA). This strategy of integrating multiple data analysis methods improved the accuracy and robustness of the study and broadened the understanding of the complex genetic background of this disease. Our cumulative findings revealed, for the first time, the possibility of LTBP1 acting as a potential key gene for OA and verified its important role in the pathogenesis of OA through the use of multiple analytical tools. Specifically, LTBP1 displayed significance in UTMOST, FUSION, and MAGMA analyses, and its potential function in the TGF-β signaling pathway, in particular, suggests that it may influence the progression of OA through mechanisms that regulate ECM formation, inflammatory response, and cartilage repair. In addition, single-cell sequencing data further revealed the specific expression patterns of LTBP1 in different cell types and the developmental trajectories, thereby providing new clues for analyzing its specific biological functions in OA.

Our study found that cells with high LTBP1 expression activate multiple signaling pathways, many of which are closely associated with OA and other fibrotic and inflammatory diseases. The ANGPTL signaling pathway is involved in endothelial cell function and has been shown to affect angiogenesis and fibrosis in OA. In our study, high expression of LTBP1 is associated with enhanced endothelial function and ECM remodeling, both of which are key features of OA pathogenesis. CypA plays a crucial role in inflammation and ECM remodeling. In OA, elevated CypA expression is linked to cartilage degradation and synovial inflammation. LTBP1 may influence inflammation and matrix degradation by regulating the CypA signaling pathway. FGF are involved in cartilage and bone repair, processes that are critical in OA. High LTBP1 expression may enhance the FGF signaling pathway, promoting chondrocyte differentiation and ECM synthesis, thereby impacting OA progression. GAS signaling is related to cellular stress responses and cell cycle regulation. Through its regulation of the TGF-β signaling pathway, LTBP1 may help maintain the balance between cell proliferation and apoptosis in OA tissues. IGFBPs regulate growth factors that are essential for cartilage health and repair. In OA, LTBP1 may influence the availability of these growth factors, particularly in chondrocytes, by modulating IGFBP activity. LIFR signaling pathway plays a role in inflammatory responses and chondrocyte survival. LTBP1’s regulation of LIFR could contribute to the recruitment of inflammatory cells, thereby exacerbating joint damage in OA. MIF is involved in inflammation and cell migration, which are crucial in OA pathogenesis. High LTBP1 expression may further drive the inflammatory environment in OA by enhancing MIF activity. PDGF is involved in synovial cell proliferation and ECM remodeling, both of which are key processes in OA progression. LTBP1’s regulation of PDGF signaling may influence the dynamics of synovial tissue in OA. PTN is involved in cell migration, growth, and angiogenesis. LTBP1 may promote tissue repair and angiogenesis in OA through PTN signaling. SPP1 is a glycoprotein involved in ECM remodeling and inflammation. LTBP1 may regulate SPP1 expression, contributing to ECM degradation and inflammation in OA. TGF-β signaling plays a central role in OA by regulating ECM synthesis and degradation. LTBP1’s direct interaction with TGF-β highlights its critical role in maintaining cartilage integrity and regulating repair mechanisms in OA. In OA, VEGF promotes cartilage vascularization, and LTBP1’s role in VEGF signaling may affect angiogenesis in OA joints. Additionally, LTBP1 may influence the inflammatory microenvironment in OA by regulating VISFATIN expression.

LTBP1 plays a critical role in the TGF-β signaling pathway, serving as a key regulator of ECM remodeling and inflammatory responses (22, 23). First, LTBP1 facilitates the activation and release of TGF-β by binding to it, which initiates a series of cellular signaling events essential for maintaining normal cell function and tissue homeostasis. In terms of ECM remodeling, LTBP1’s role primarily involves regulating the synthesis and degradation of ECM components, thus influencing cell adhesion, migration, and proliferation. Through these mechanisms, LTBP1 is not only crucial in tissue repair and regeneration but also implicated in various pathological conditions, particularly in chronic inflammation and fibrosis. Additionally, LTBP1 may promote local inflammatory responses by modulating the infiltration and activation of inflammatory cells (24, 25). Therefore, a deeper exploration of LTBP1’s functions within the TGF-β signaling pathway, along with its broader biological roles in ECM remodeling and inflammation, will enhance our understanding of its significance in various physiological and pathological states (26–28). It has been shown that LTBP1 has an important impact on the development of a variety of diseases through the binding to and modulation of TGF-β. For example, in fibrotic diseases, LTBP1 accelerates fibrosis by promoting TGF-β activation (29). In cancer studies, LTBP1 is highly expressed in esophageal squamous cell carcinoma (ESCC) tissues, and its overexpression is positively correlated with lymph node metastasis. Functional experiments have shown that the knockdown of LTBP1 inhibited the invasive and migratory abilities of ESCC cells and decreased the epithelial-mesenchymal transition and cancer-associated fibroblast transformation, suggesting an oncogenic role of LTBP1 in ESCC progression (30). Some studies have also explored the potential bridging role of LTBP1 between depression and glioblastoma and found that LTBP1 affects glioblastoma and depression/anxiety disorders by regulating the organization and function of the ECM. Also, glioblastoma cells with high LTBP1 expression have a significantly greater proliferation and migration capacity, making it an important molecule that influences the prognosis of glioblastoma patients (31). In OA, the TGF-β signaling pathway has long been shown to play an important role in cartilage ECM maintenance and cartilage repair (32–34). However, the specific mechanism regarding the role of LTBP1 in the pathogenesis of OA remains unclear. In the present study, we combined the results of the analysis at the trans-tissue and single-cell levels and clarified that the high expression of LTBP1 is closely related to the activation of the TGF-β signaling pathway. We further found that LTBP1 not only displayed significant expression differences in different types of chondrocytes but also played an important role in the cell differentiation trajectory and signaling pathway regulation. These results suggest that LTBP1 may influence the balance of cartilage degradation and repair by regulating the activity of the TGF-β signaling pathway, thereby driving OA pathogenesis. This finding provides a theoretical basis for the further exploration of the molecular function of LTBP1 and suggests its potential as a diagnostic marker or therapeutic target. This study not only fills the gap in the research related to LTBP1 and OA but also indicates the direction for future functional validation experiments and targeted therapy development.

In this study, through single-cell sequencing analysis, we identified multiple cell types, including FC, HTC, RepC, and RegC, closely related to OA pathogenesis. FC exhibited high LTBP 1 expression in OA samples, suggesting their important role in OA progression, especially through promoting the formation and regeneration of the ECM. HTC are a type of chondrocyte found in a degenerative and proliferative state within the cartilage, commonly observed in the pathological process of OA. The role of HTC in OA cartilage is closely related to cell proliferation and differentiation, and they regulate cell function and survival through the notch signaling pathway. RepC exhibit regenerative potential and are primarily involved in the repair process of cartilage in OA. RegC typically play a role in maintaining immune homeostasis within tissues and regulate immune responses to either promote or inhibit the progression of the disease. Furthermore, through pseudotime trajectory analysis, we identified five major”states”of OA cells, representing different developmental and differentiation stages in the OA progression. Specifically, state 1 predominantly consists of HomC with high LTBP1 expression, which may be involved in the early stages of OA and contribute to local repair. States 2, 3, and 4, which are enriched in RepC, are associated with the repair processes and cellular reprogramming in OA. State 5, consisting mainly of FC, may play a crucial role in ECM remodeling during the chronic phase of OA. The functional differences of cells across these states provide new insights into the mechanisms underlying OA progression, particularly in the dynamic balance between repair and degeneration. The results in **Figure 6** further highlight this cell-to-cell communication pattern, especially in the LTBP1 high-expression group, where FC and HTC act as”sender”and “receiver”, respectively. This classification not only reveals the diverse roles that different cell types play in OA development but also suggests potential therapeutic targets, especially in the regulation of cell signaling and intercellular communication mechanisms, thereby providing new directions for future therapeutic strategies for OA. Overall, these results suggest that intercellular communication and LTBP1 signaling regulation play a central role in OA pathogenesis. Future studies should explore the specific roles of these cell types and signaling pathways in OA and their impact on the potential therapeutic strategies.

This study demonstrated significant novelty and methodological advantages over previous OA-related gene studies. Previous studies, mostly based on GWAS, have identified several key OA-related genes, such as GDF5, RUNX2, and SMAD3 (35–37). However, these studies have mainly focused on the association signals at a single gene locus, making it difficult to directly reveal the functional genes corresponding to these signals and their roles in specific tissues. Unlike the traditional GWAS methods, this study systematically identified and validated the potential role of LTBP1 as an OA candidate gene for the first time through cross-tissue TWAS analysis and functional verification at the single-tissue level. As a key regulator of the TGF-β signaling pathway, LTBP1 is directly involved in the regulation of the ECM and the balance of chondrocyte differentiation and repair (38). In this study, we found that LTBP1 not only exhibited significant cross-tissue association in multiple tissues but also further confirmed its differential expression and functional specificity in different chondrocyte subtypes through single-cell sequencing data for the first time. The application of cross-tissue TWAS compensates for the limitations of traditional GWAS studies and enables the integration of gene expression signatures from different tissues to more comprehensively capture the complex genetic mechanisms of diseases (12, 39). Single-tissue analysis, on the other hand, further focuses on specific tissues (e.g., articular cartilage) to reveal the precise roles of genes under certain pathological conditions. The combination of the two provides a new perspective for exploring the multitissue and multiscale genetic basis of complex diseases. In addition, by integrating LTBP1 with GWAS results, this study not only verified its prominence as a candidate gene but also improved the understanding of biological signals.

While our study provides novel insights into the role of LTBP1 in OA through the integration of multiple data analysis methods, we acknowledge the limitations inherent to the TWAS approach. First, while we utilized multiple tissues and single-tissue TWAS analyses, the reference eQTL data are limited to European populations, which may affect the generalizability of the results to other ethnicities. Moreover, gene expression data collected from public datasets such as GTEx may not fully represent the specific pathological conditions of OA, as these datasets are typically derived from healthy tissues. Future studies should incorporate datasets from OA-specific tissues to better reflect disease-specific gene expression patterns. Additionally, the cross-tissue nature of TWAS has the potential to overlook tissue-specific regulatory mechanisms that could be crucial in disease pathogenesis. The current study’s lack of experimental validation is a significant limitation that may affect the reliability of the findings and the validity of the conclusions. Therefore, future research must place more emphasis on functional validation to provide a solid empirical foundation. Specifically, adopting a CRISPR-mediated LTBP1 gene knockout model would be a promising direction. This model enables precise editing of the LTBP1 gene, allowing us to observe its role in relevant biological processes. Not only will this approach validate theoretical hypotheses, but it will also deepen our understanding of the functional significance of LTBP1 within specific biological pathways. Through such experimental design, we aim to provide more compelling evidence to clarify the biological importance of LTBP1, thereby advancing further developments in this field.

## 5 Conclusion

In this study, the role of LTBP1 as a key candidate gene for OA was identified and validated for the first time by cross-tissue and single-tissue transcriptome association analyses and single-cell sequencing. These results provide an important basis for the potential of LTBP1 as a diagnostic marker and therapeutic target and indicate the direction for future research and application.

## Data Availability

The datasets presented in this study can be found in online repositories. The names of the repository/repositories and accession number(s) can be found in the article/supplementary material.

## Supplementary material

Supplementary Figure 1 Pseudotime trajectory analysis of OA cells. **(A)**. Developmental trajectory of the top 6 DEGs. **(B)**. Dot plot of the top 6 DEGs. **(C)**. Heatmap of the top 50 DEGs. **(D)**. Developmental trajectories of different samples. **(E)**. Developmental trajectory of LTBP1.

Supplementary Figure 2 The communication patterns between the seven cell types in the LTBP1 high-expression OA cells. **(A)**. The communication patterns of receivers. **(B)**. The communication patterns of senders. **(C)**. The dot plot for the receivers communication patterns of the seven cell types. **(D)**. The dot plot for the senders communication patterns of the seven cell types. **(E)**. The Sankey plot for the receivers communication patterns of the seven cell types. **(F)**. The Sankey plot for the senders communication patterns of the seven cell types. **(G)**. The dot plot for all receiver and sender pairings.

Supplementary Figure 3 The communication patterns between the seven cell types in the LTBP1 low-expression OA cells. **(A)**. The communication patterns of receivers. **(B)**. The communication patterns of senders. **(C)**. The dot plot for the receivers communication patterns of the seven cell types. **(D)**. The dot plot for the senders communication patterns of the seven cell types. **(E)**. The Sankey plot for the receivers communication patterns of the seven cell types. **(F)**. The Sankey plot for the senders communication patterns of the seven cell types. **(G)**. The dot plot for all receiver and sender pairings.

Supplementary Figure 4 Cellular interaction of OA cells with low LTBP1 expression. The cellular interaction network identified cell clusters in various signaling pathways, including **(A)**. ANGPTL, **(B)**. CypA, **(C)**. FGF, **(D)**. IGFBP, **(E)**. MIF, **(F)**. SPP1, **(G)**. TGF-β, and **(H)**. VISFATIN pathways.
