# Supplementary figures and images for: A splicing-based multitissue association study of joint transcriptomes identified susceptibility genes for osteoarthritis
Source: Front Immunol. 2025 Sep 11;16:1590008. doi: 10.3389/fimmu.2025.1590008 (PMC12460263; doi:10.3389/fimmu.2025.1590008)

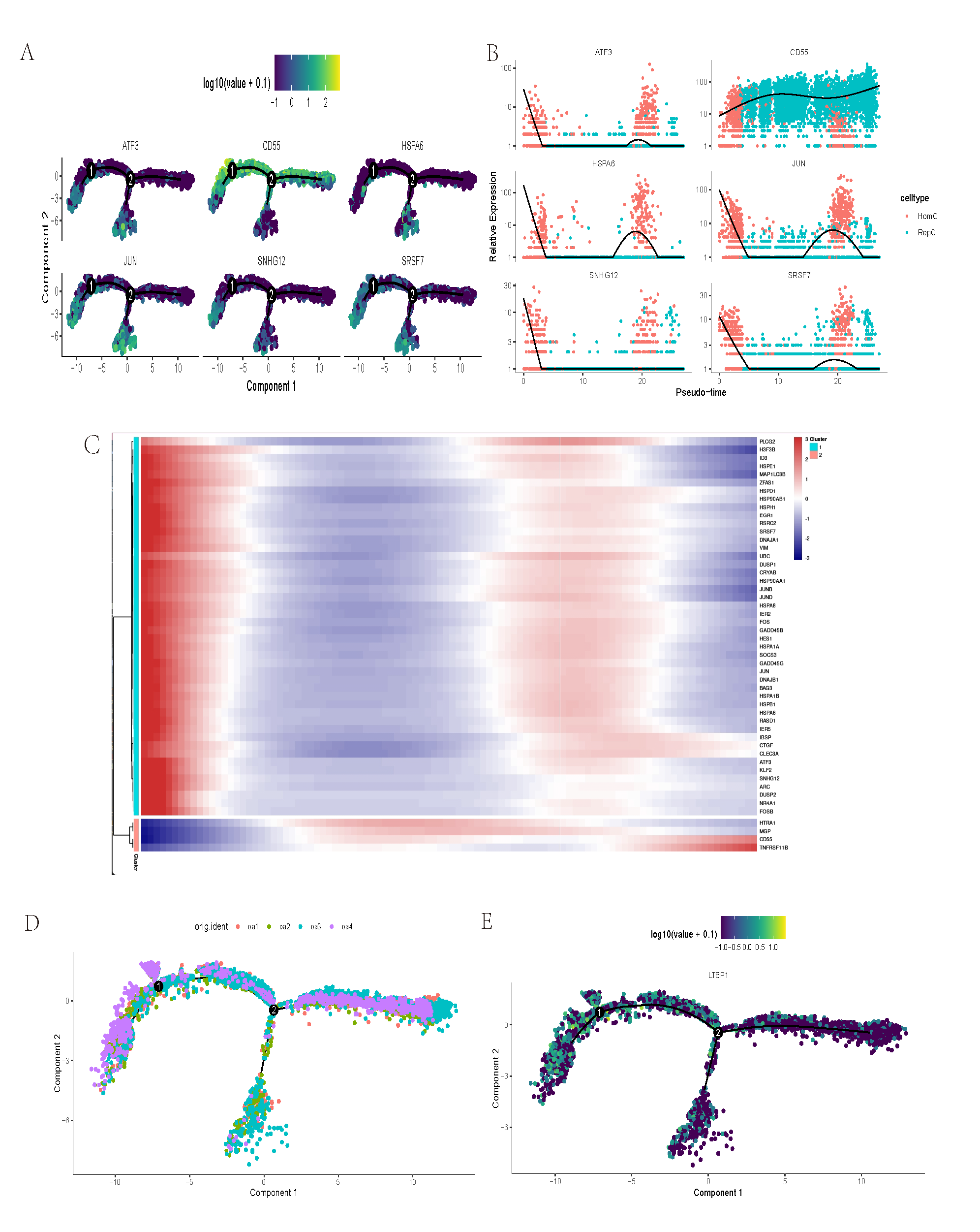

Supplement: Supplementary Figure 1 — Pseudotime trajectory analysis of OA cells. (A). Developmental trajectory of the top 6 DEGs. (B). Dot plot of the top 6 DEGs. (C). Heatmap of the top 50 DEGs. (D). Developmental trajectories of different samples. (E). Developmental trajectory of LTBP1. [file Image1.tif]

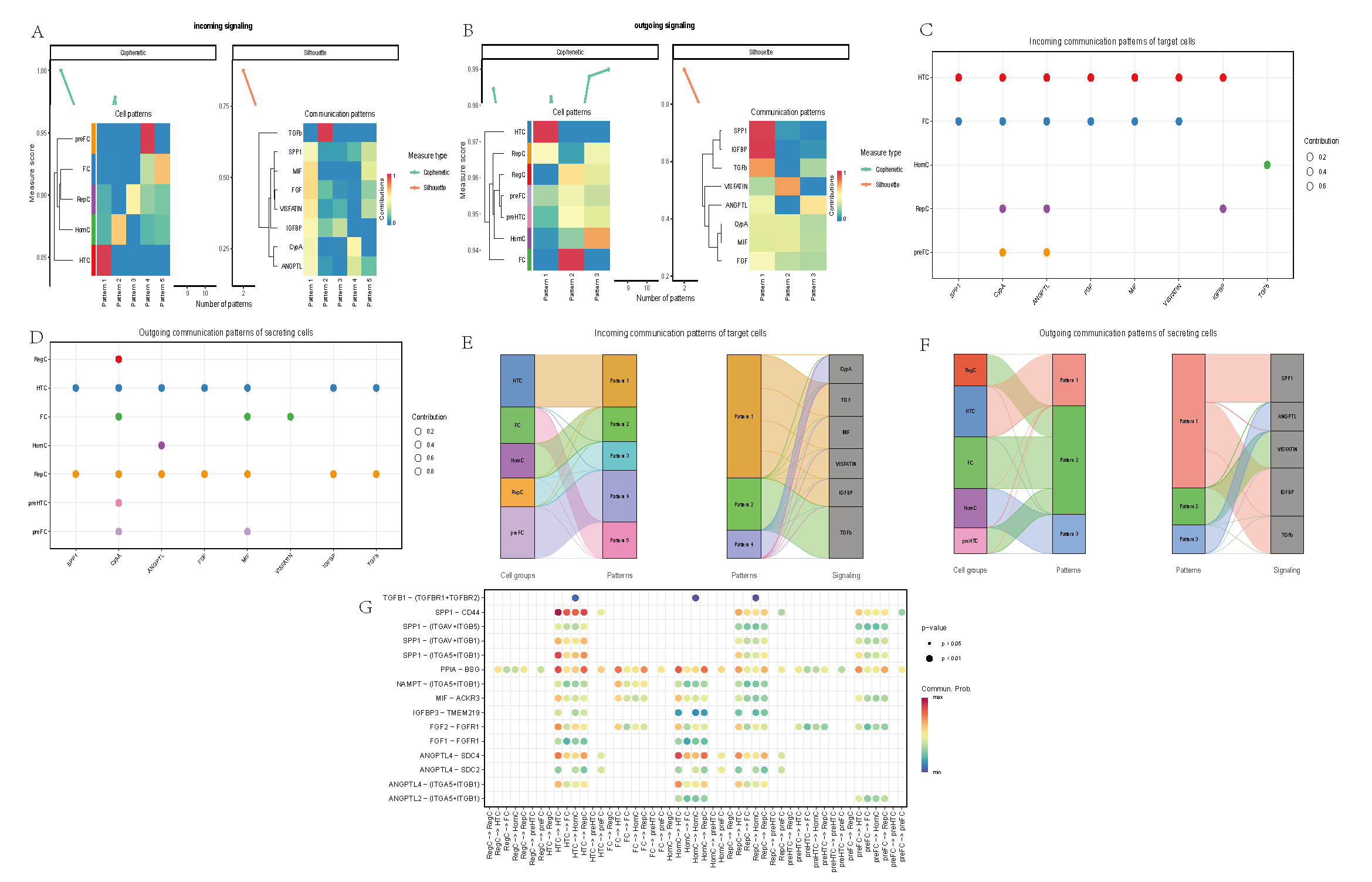

Supplement: Supplementary Figure 2 — The communication patterns between the seven cell types in the LTBP1 high-expression OA cells. (A). The communication patterns of receivers. (B). The communication patterns of senders. (C). The dot plot for the receivers communication patterns of the seven cell types. (D). The dot plot for the senders communication patterns of the seven cell types. (E). The Sankey plot for the receivers communication patterns of the seven cell types. (F). The Sankey plot for the senders communication patterns of the seven cell types. (G). The dot plot for all receiver and sender pairings. [file Image2.tif]

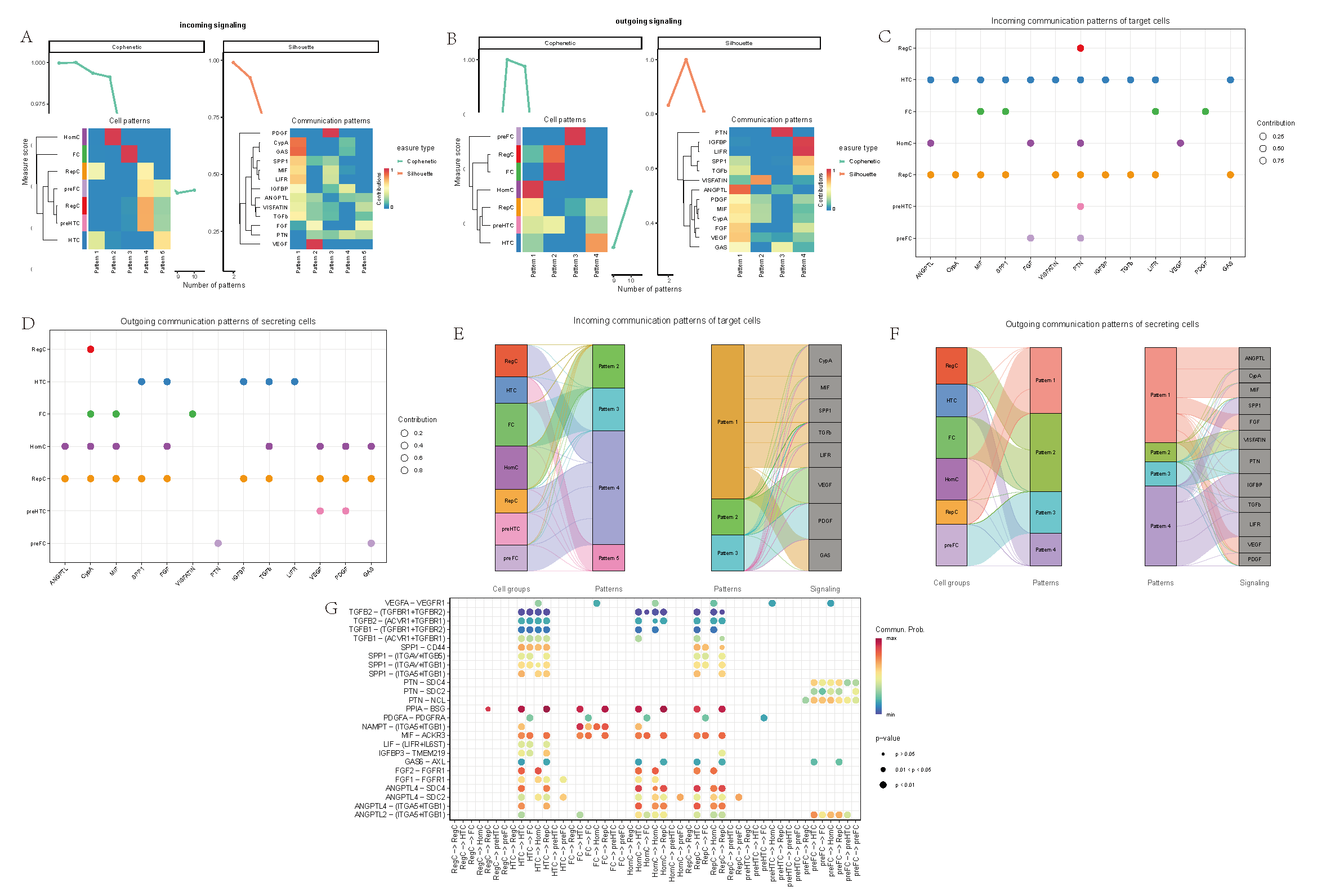

Supplement: Supplementary Figure 3 — The communication patterns between the seven cell types in the LTBP1 low-expression OA cells. (A). The communication patterns of receivers. (B). The communication patterns of senders. (C). The dot plot for the receivers communication patterns of the seven cell types. (D). The dot plot for the senders communication patterns of the seven cell types. (E). The Sankey plot for the receivers communication patterns of the seven cell types. (F). The Sankey plot for the senders communication patterns of the seven cell types. (G). The dot plot for all receiver and sender pairings. [file Image3.tif]

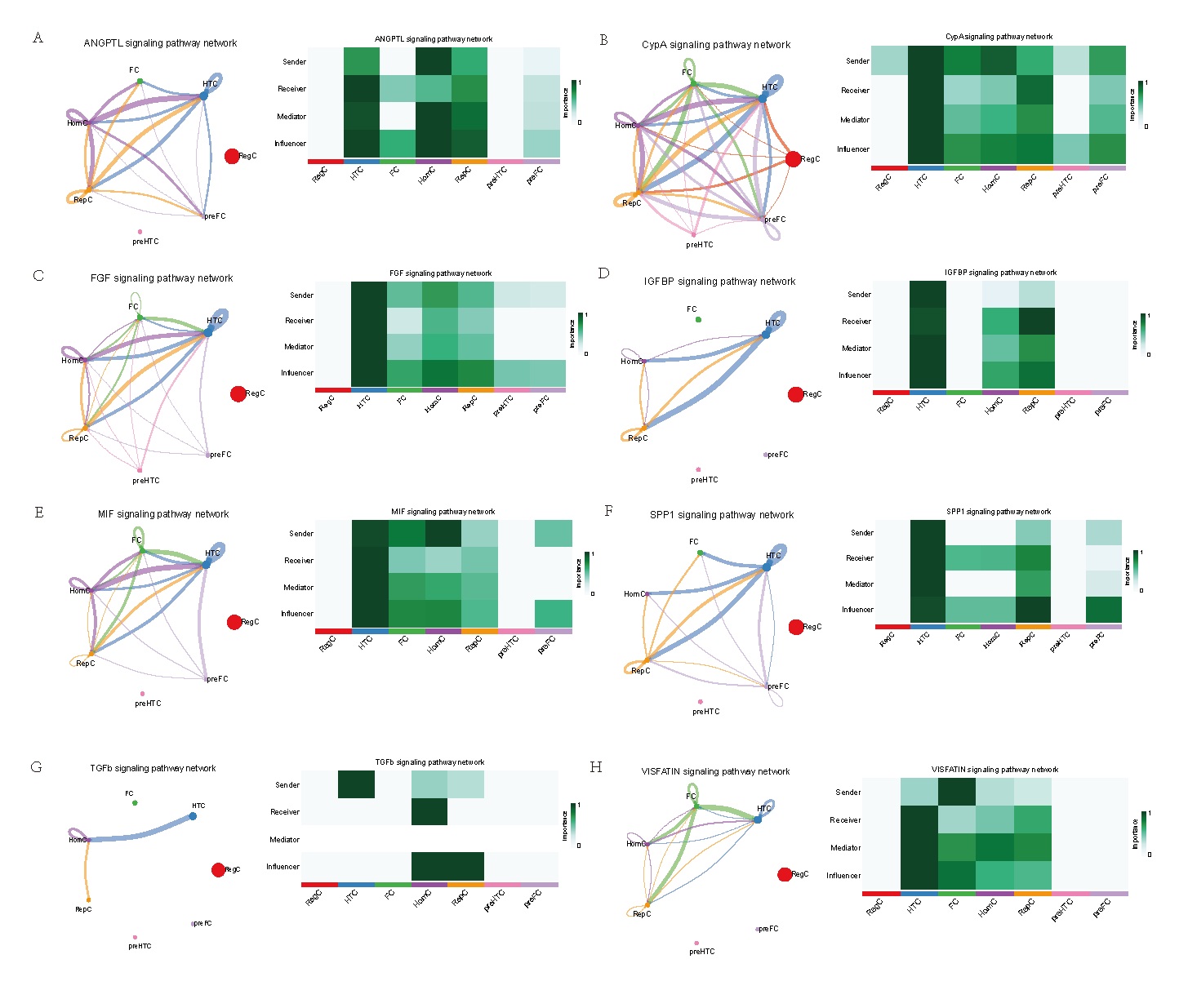

Supplement: Supplementary Figure 4 — Cellular interaction of OA cells with low LTBP1 expression. The cellular interaction network identified cell clusters in various signaling pathways, including (A). ANGPTL, (B). CypA, (C). FGF, (D). IGFBP, (E). MIF, (F). SPP1, (G). TGF-β, and (H). VISFATIN pathways. [file Image4.tif]
