# Supplementary material for: A splicing-based multitissue association study of joint transcriptomes identified susceptibility genes for osteoarthritis
Source: Front Immunol. 2025 Sep 11;16:1590008. doi: 10.3389/fimmu.2025.1590008 (PMC12460263; doi:10.3389/fimmu.2025.1590008)
Supplement: Supplementary file 5 [file Table1.docx]

Table S1. The results of TWAS analyses in cross-tissue.

| Ensemeble ID | Test_score | p_value | Gene symbole | CHR | start_hg19 | end_hg38 | FDR |
| --- | --- | --- | --- | --- | --- | --- | --- |
| ENSG00000013810 | 8.448541495 | 7.58E-05 | TACC3 | 4 | 1712858 | 1745171 | 0.018944407 |
| ENSG00000049323 | 14.87417626 | 1.17E-07 | LTBP1 | 2 | 32946953 | 33399509 | 0.000146493 |
| ENSG00000068078 | 8.716800064 | 0.000136993 | FGFR3 | 4 | 1793293 | 1808872 | 0.025175506 |
| ENSG00000068912 | 8.40401036 | 0.000152633 | ERLEC1 | 2 | 53787009 | 53833038 | 0.025175506 |
| ENSG00000091106 | 11.98420645 | 1.83E-06 | NLRC4 | 2 | 32224453 | 32265732 | 0.001143939 |
| ENSG00000109705 | 14.48779456 | 3.38E-07 | NKX3-2 | 4 | 13540830 | 13544508 | 0.000279133 |
| ENSG00000115239 | 205.6288067 | 5.39E-05 | ASB3 | 2 | 53532672 | 53860160 | 0.014422058 |
| ENSG00000115419 | 8.875635048 | 0.000136896 | GLS | 2 | 190880821 | 190965552 | 0.025175506 |
| ENSG00000119737 | 9.59926901 | 9.25E-05 | GPR75 | 2 | 53852912 | 53859967 | 0.021078675 |
| ENSG00000124374 | 21.97053072 | 1.38E-10 | PAIP2B | 2 | 71182738 | 71227103 | 5.16E-07 |
| ENSG00000137601 | 7.918155268 | 0.000154533 | NEK1 | 4 | 169369704 | 169612632 | 0.025175506 |
| ENSG00000163235 | 10.76108869 | 1.50E-05 | TGFA | 2 | 70447284 | 70554193 | 0.006259709 |
| ENSG00000169564 | 362.213346 | 1.06E-09 | PCBP1 | 2 | 70087477 | 70089203 | 1.98E-06 |
| ENSG00000174137 | 10.12258187 | 1.95E-05 | FAM53A | 4 | 1617915 | 1684313 | 0.006657987 |
| ENSG00000179915 | 10.23970481 | 2.55E-05 | NRXN1 | 2 | 49918503 | 51225575 | 0.007955901 |
| ENSG00000184470 | 7.892185392 | 0.000245414 | TXNRD2 | 22 | 19875517 | 19941820 | 0.038315232 |
| ENSG00000204792 | 7.474471796 | 9.56E-05 | LINC01291 | 2 | 74918148 | 74938418 | 0.021078675 |
| ENSG00000211678 | 11.56603771 | 1.82E-05 | IGLJ3 | 22 | 22904850 | 22905025 | 0.006657987 |
| ENSG00000229204 | 237.6555947 | 9.58E-06 | PTGES3P3 | 4 | 169791221 | 169791702 | 0.004484931 |
| ENSG00000231327 | 313.1918885 | 3.72E-07 | LINC01816 | 2 | 70124034 | 70132923 | 0.000279133 |
| ENSG00000246095 | 9.899617356 | 3.71E-05 | LINC01096 | 4 | 13546075 | 13547801 | 0.010695071 |
| ENSG00000248399 | 11.61266396 | 8.48E-06 | RPL7P57 | 4 | 2463797 | 2464124 | 0.004484931 |
