# Supplementary material for: A splicing-based multitissue association study of joint transcriptomes identified susceptibility genes for osteoarthritis
Source: Front Immunol. 2025 Sep 11;16:1590008. doi: 10.3389/fimmu.2025.1590008 (PMC12460263; doi:10.3389/fimmu.2025.1590008)
Supplement: Supplementary file 6 [file Table2.docx]

Table S2. FUSION identified 21 significant genes associated with osteoarthritis.

| Group | Gene symbole | TWAS.Z | TWAS.P | JOINT.BETA | JOINT.BETA.SE | JOINT.Z | JOINT.P | COND.BETA | COND.BETA.SE | COND.Z | COND.P |
| --- | --- | --- | --- | --- | --- | --- | --- | --- | --- | --- | --- |
| GTExv8.ALL.Cells_Cultured_fibroblasts | KHK | -3.28 | 0.00104 |  |  |  |  | -0.134 | 0.392 | -0.341 | 0.733 |
| GTExv9.ALL.Cells_Cultured_fibroblasts | LYG1 | 3.54 | 0.000393 |  |  |  |  | 0.19 | 0.448 | 0.425 | 0.671 |
| GTExv10.ALL.Cells_Cultured_fibroblasts | EIF5B | 3.61 | 0.000304 |  |  |  |  | 1.111 | 0.746 | 1.491 | 0.136 |
| GTExv12.ALL.Cells_Cultured_fibroblasts | LTBP1 | -5.47 | 4.62E-08 | -5.47 | 1 | -5.47 | 4.62E-08 |  |  |  |  |
| GTExv13.ALL.Cells_Cultured_fibroblasts | HDLBP | -4.19 | 2.76E-05 | -4.19 | 1 | -4.19 | 2.76E-05 |  |  |  |  |
| GTExv14.ALL.Cells_Cultured_fibroblasts | REV1 | -3.75 | 0.000176 | -3.75 | 1 | -3.75 | 0.000176 |  |  |  |  |
| GTExv15.ALL.Cells_Cultured_fibroblasts | CGREF1 | -3.42 | 0.000629 | -3.42 | 1 | -3.42 | 0.000629 |  |  |  |  |
| GTExv16.ALL.Cells_Cultured_fibroblasts | CARF | 3.36 | 0.000775 | 3.36 | 1 | 3.36 | 0.000775 |  |  |  |  |
| GTExv17.ALL.Cells_Cultured_fibroblasts | MGAT5 | 3.38 | 0.000727 | 3.38 | 1 | 3.38 | 0.000727 |  |  |  |  |
| GTExv18.ALL.Cells_Cultured_fibroblasts | PUS10 | 3.33 | 0.000879 | 3.33 | 1 | 3.33 | 0.000879 |  |  |  |  |
| GTExv19.ALL.Cells_Cultured_fibroblasts | ACYP2 | 3.34 | 0.00085 | 3.34 | 1 | 3.34 | 0.00085 |  |  |  |  |
| GTExv20.ALL.Cells_Cultured_fibroblasts | CYRIA | 3.53 | 0.000411 | 3.53 | 1 | 3.53 | 0.000411 |  |  |  |  |
| GTExv21.ALL.Cells_Cultured_fibroblasts | LINC01291 | -3.64 | 0.000276 | -3.64 | 1 | -3.64 | 0.000276 |  |  |  |  |
| GTExv22.ALL.Cells_Cultured_fibroblasts | VIT | -3.57 | 0.000358 | -3.57 | 1 | -3.57 | 0.000358 |  |  |  |  |
| GTExv23.ALL.Cells_Cultured_fibroblasts | RPL7P57 | 3.34 | 0.000824 | 3.34 | 1 | 3.34 | 0.000824 |  |  |  |  |
| GTExv24.ALL.Cells_Cultured_fibroblasts | ZDHHC8 | -4.22 | 2.45E-05 | -4.22 | 1 | -4.22 | 2.45E-05 |  |  |  |  |
| GTExv25.ALL.Cells_Cultured_fibroblasts | FGFR3 | -3.79 | 0.000152 |  |  |  |  | -1.241 | 0.917 | -1.3539 | 0.176 |
| GTExv26.ALL.Cells_Cultured_fibroblasts | FAM53A | -5.05 | 4.31E-07 |  |  |  |  | -0.0333 | 0.491 | -0.0678 | 0.946 |
| GTExv27.ALL.Cells_Cultured_fibroblasts | CLCN3 | -3.66 | 0.000254 | -3.66 | 1 | -3.66 | 0.000254 |  |  |  |  |
| GTExv28.ALL.Cells_Cultured_fibroblasts | PRDM5 | -3.67 | 0.00024 | -3.67 | 1 | -3.67 | 0.00024 |  |  |  |  |
| GTExv29.ALL.Cells_Cultured_fibroblasts | SLBP | -5.54 | 3.05E-08 | -4.15 | 1.06 | -3.91 | 9.12E-05 |  |  |  |  |
